# Left Atrial Appendage Closure for Primary and Secondary Stroke Prevention in Patients With Hypertrophic Cardiomyopathy and Atrial Fibrillation: A Pilot Study

**DOI:** 10.3389/fcvm.2021.719755

**Published:** 2021-10-13

**Authors:** Bin-Feng Mo, Rui Zhang, Jia-Li Yuan, Jian Sun, Peng-Pai Zhang, Wei Li, Mu Chen, Xing-Xing Cai, Yi-Chi Yu, Qun-Shan Wang, Yi-Gang Li

**Affiliations:** Department of Cardiology, Xinhua Hospital Affiliated to Shanghai Jiao Tong University School of Medicine, Shanghai, China

**Keywords:** hypertrophic cardiomyopathy, atrial fibrillation, left atrial appendage closure, stroke prevention, anticoagulation

## Abstract

**Background:** The aim of this study was to investigate the efficacy of left atrial appendage closure (LAAC) for primary and secondary stroke prevention in patients with hypertrophic cardiomyopathy (HCM) and atrial fibrillation (AF).

**Methods:** This pilot study enrolled 36 patients with HCM and AF who underwent LAAC between April 2017 and December 2019, of whom 22 were for primary stroke prevention and 14 were for secondary prevention.

**Results:** The patients enrolled in this study had non-obstructive (86.1%) or mild obstructive (13.9%) HCM. Patients in the Secondary Prevention Group had higher CHA2DS2-VASc scores (5.1 ± 1.4 vs. 2.6 ± 1.6, *P* < 0.001) and higher HAS-BLED scores (2.8 ± 1.0 vs. 1.5 ± 0.9, *P* < 0.001) compared with those in the Primary Prevention Group. Successful closure with satisfactory seals (residual leak ≤ 5 mm) was achieved in all patients, with complete occlusion in 86.4% of the Primary Prevention Group and 92.9% of the Secondary Prevention Group. Procedural-related complications included one pericardial effusion and one groin hematoma. One device-related thrombus was identified in the Secondary Prevention Group and resolved after anticoagulation. During a mean follow-up time of 28.4 months, one bleeding event was recorded. There were no thromboembolic events or deaths in either group, with 97.2% of the patients achieving freedom from anticoagulation therapy.

**Conclusions:** Initial results suggest that LAAC can be a safe and feasible alternative for primary and secondary stroke prevention in selected patients with HCM and AF. Further studies with larger samples are required.

## Introduction

Atrial fibrillation (AF) is the most common sustained arrhythmia in patients with hypertrophic cardiomyopathy (HCM), with an estimated prevalence of 20–30% (1, 2). Patients with concomitant AF and HCM are at a high incidence of stroke regardless of the CHA2DS2-VASc score. Current guidelines recommend lifelong anticoagulation for all HCM patients who develop AF (3, 4).

Percutaneous left atrial appendage closure (LAAC), especially with WATCHMAN device, has been demonstrated in randomized trials to reduce stroke and therefore can be an alternative to warfarin therapy for stroke prevention in the general AF population (5, 6). The efficacy of LAAC for stroke prevention in patients with HCM and AF, however, has not been studied, although the incidences of left atrial appendage (LAA) thrombus in this subpopulation has been reported to be 7.1–10.7% (7, 8). Therefore, the aim of this pilot study was to evaluate the efficacy of LAAC in patients with HCM and AF for primary and secondary stroke prevention.

## Methods

### Study Population

This retrospective, single-center study included consecutive patients with HCM and non-valvular AF who underwent LAAC between April 2017 and December 2019. Inclusion criteria: patients were over 18 years, presented with HCM and non-valvular AF, and were complicated with at least one of the following situations: (a) a high bleeding risk with HAS-BLED score ≥ 3; (b) an unwillingness to receive regular oral anticoagulation (OAC) therapy; (c) intolerance to chronic OAC; (d) stroke or thromboembolism even under OAC treatment. The diagnosis of HCM was based on a two-echocardiographic demonstration of a hypertrophied and non-dilated left ventricle (wall thickness ≥ 15 mm), in the absence of another cardiac or systemic disease capable of producing a similar magnitude of hypertrophy (9, 10). Documentation of AF was based on an electrocardiogram or Holter obtained either after the acute onset of symptoms or during a routine examination. The exclusive criteria included severe left ventricular outflow tract obstruction needing surgery or alcohol septal ablation, intraventricular obstruction, ventricular aneurysm, previous alcohol septal ablation or surgical myectomy due to outflow tract obstruction, and requirement for long-term anticoagulation therapy for reasons other than AF.

The retrospective study was approved by the Ethics Committee of Xinhua Hospital Affiliated to Shanghai Jiao Tong University School of Medicine and complies with the Declaration of Helsinki. Written informed consent was obtained from each patient.

### LAAC Procedure

Transesophageal echocardiography (TEE) was performed prior to the procedure to rule out LAA thrombi. A cardiac computed tomography (CT) scan and 3-dimensional reconstruction of the left atrium were available in 90.6% (29/32) of the patients for preprocedure planning.

The LAAC procedure was performed as described previously (11). In brief, the procedure was performed under local anesthesia and fluoroscopy guidance, and TEE was introduced under deep sedation after device deployment to reconfirm the position of the device before release. A mean left atrial pressure above 10 mmHg was obtained after transseptal puncture. A Watchman device (Boston Scientific, Marlborough, MA, USA) with appropriate size (21, 24, 27, 30, and 33 mm) was chosen, generally 10–30% oversizing based on the ostial width of the LAA measured by angiography or cardiac CT. The device was then advanced into the delivery sheath and deployed by sheath retraction guided by fluoroscopy. Preliminary assessment was performed by angiography and tug test under fluoroscopy to check the device position and stability. TEE was then performed to reconfirm the position with minimal (<5 mm) to no residual peri-device leaks and a proper compression ratio under deep sedation. The device was released if it was verified by the assessment of “PASS” criteria.

### Post-procedural Management and Follow-Up

Post discharge, office or transtelephonic visits were scheduled at the 3, 6, and 12 months following the procedure and once half a year thereafter. Adverse events reported during follow-up visit, based on the Percutaneous LAA occlusion Munich Consensus Document (12), including mortality, thromboembolic events (stroke and systemic embolism) and bleeding. TEE was performed to assess device occlusion safety and efficiency, including peri-device flow, device-related thrombus (DRT), device embolism, and pericardial effusion at 45 days and 6 months of follow-up time points. At discharge, OAC with Vitamin K antagonist or novel oral anticoagulant (NOAC) was recommended for all patients for at least 45 days unless there were contraindications. Dual antiplatelet therapy (aspirin and clopidogrel) was recommended for another 4.5 months and then life-long aspirin was prescribed if follow-up TEE confirmed satisfactory seal (jet <5 mm in width). If unsatisfactory seal or DRT was detected, OAC was continued until satisfactory seal was achieved or DRT was resolved by the evaluation of TEE repeated every 3–6 months.

### Statistical Analysis

Continuous variables were described as mean ± standard deviation (median [interquartile range] for non-normal data) and compared using Student's *t*-test (Mann–Whitney U test if normality not satisfied). Categorical variables were presented as percentages and analyzed using chi-square test or Fisher exact test where appropriate. All analyses were performed using SPSS version 22.0 (IBM Software Inc., Armonk, NY, USA). Two-sided *P*-values of <0.05 were considered statistically significant.

## Results

### Baseline Characteristics

Data from 837 patients who underwent LAAC between April 2017 and December 2019 was reviewed. Thirty-nine patients who were diagnosed with HCM and AF were identified. Two patients with severe left ventricular outflow tract obstruction and one patient with intraventricular obstruction and apical aneurysm were excluded. In total, 36 cases with HCM and AF who underwent LAAC were included in this study, of whom 22 were for primary stroke prevention and 14 were for secondary prevention. Indications for LAAC included high bleeding risk (33.3%), unwilling to take OAC (47.2%), intolerance to chronic OAC (13.9%) and stroke or thromboembolism even under OAC treatment (5.6%).

Baseline clinical and demographic characteristics are presented in Table 1. The mean age was 68.8 ± 8.3 in the Primary Prevention Group and 69.5 ± 7.1 in the Secondary Prevention Group. Women composed 36.4% and 35.7% of the two groups, respectively. Seven patients (31.8%) in the Primary Prevention Group and six patients (42.9%) in the Secondary Prevention Group had a prior failed AF ablation. There were no significant differences in comorbidities, except prior stroke. Patients in the Secondary Prevention Group had higher CHA2DS2-VAS scores (5.1 ± 1.4 vs. 2.6 ± 1.6, *P* < 0.001) and higher HAS-BLED scores (2.8 ± 1.0 vs. 1.5 ± 0.9, *P* < 0.001) compared with those in the Primary Prevention Group.

**Table 1:** Baseline characteristics of patients with hypertrophic cardiomyopathy and atrial fibrillation.

	**Primary prevention *N* = 22**	**Secondary prevention*N* = 14**	***P*-value**
Age (years)	68.8 ± 8.3	69.5 ± 7.1	0.801
Female	8 (36.4)	5 (35.7)	0.968
AF duration (years)	6.0 ± 3.4	8.2 ± 6.6	0.208
Paroxysmal AF	7 (31.8)	3 (21.4)	0.497
Persistent AF	15 (68.2)	11 (78.6)	0.497
Prior failed AF ablation	7 (31.8)	6 (42.9)	0.501
Comorbidity			
Hypertension	13 (59.1)	11 (78.6)	0.227
Heart failure	6 (27.3)	4 (28.6)	0.932
Diabetes mellitus	6 (27.3)	5 (35.7)	0.592
Prior stroke	0 (0.0)	14 (100.0)	
Coronary artery disease	5 (22.7)	6 (42.9)	0.201
Chronic kidney disease	2 (9.1)	4 (28.6)	0.126
With pacemakers	3 (13.6)	2 (14.3)	0.956
With ICDs	1 (4.5)	0 (0.0)	0.425
CHA_2_DS_2_-VASc score	2.6 ± 1.6	5.1 ± 1.4	<0.001
HAS-BLED score	1.5 ± 0.9	2.8 ± 1.0	<0.001
Echocardiography			
LAD (mm)	47.1 ± 5.5	50.0 ± 5.8	0.160
LVEDD (mm)	49.4 ± 4.7	48.6 ± 8.8	0.724
LVEF (%)	60.1 ± 6.5	63.2 ± 4.0	0.113
Max LV thickness (mm)	19.5 ± 3.1	20.5 ± 4.1	0.450
Rest LVOTG ≥ 30 mmHg	2 (9.1)	2 (14.3)	0.629
Non-obstructive	19 (86.4)	12 (85.7)	0.956
Obstructive	3 (13.6)	2 (14.3)	0.956
SAM	2 (9.1)	3 (21.4)	0.297

*Values presented are n (%) or mean ± standard deviation as appropriate. AF, atrial fibrillation; ICD, implantable cardioverter defibrillator; LAD, left atrial diameter; LV, left ventricular; LVEDD, left ventricular end-diastolic diameter; LVEF, left ventricular ejection fraction; LVOTG, left ventricular outflow tract gradient; SAM, systolic anterior motion*.

The parameters of ultrasound cardiography showed no differences between the two groups (left atrial diameter, 47.1 ± 5.5 mm vs. 50.0 ± 5.8 mm; ejection fraction, 60.1 ± 6.5% vs. 63.2 ± 4.0%; max wall thickness, 19.5 ± 3.1 mm vs. 20.5 ± 4.1 mm; rest left ventricular outflow tract gradient ≥30 mmHg, 9.1% vs. 14.3%). The majority of patients had non-obstructive HCM (86.4 and 85.7% in the two groups, respectively).

### Procedural Characteristics and Safety

The procedural characteristics are demonstrated in Table 2. Successful closure with satisfactory seals (residual leak ≤ 5 mm) was achieved in all patients, with mean procedure time of 53.4 ± 6.7 min and 52.1 ± 7.4 min for the Primary and Secondary Prevention Groups, respectively. Complete occlusion was achieved in 86.4% of the Primary Prevention Group and 92.9% of the Secondary Prevention Group. The median number of devices used was 1 (1.0–1.0), with mean device sizes of 28.7 ± 4.1 mm in the Primary Prevention Group and 29.6 ± 3.3 mm in the Secondary Prevention Group.

**Table 2:** Procedural characteristics and safety.

	**Primary prevention *N* = 22**	**Secondary prevention *N* = 14**	***P*-value**
Procedure time (min)	53.4 ± 6.7	52.1 ± 7.4	0.578
Fluoroscopy time (min)	7.4 ± 2.6	6.7 ± 2.1	0.350
Left atrial pressure (mmHg)	23.1 ± 5.5	22.9 ± 7.2	0.926
Successful implantation	22 (100.0)	14 (100.0)	
LAA ostium width (mm)	23.0 ± 4.0	24.2 ± 3.6	0.376
Number of devices used	1.0 (1.0–1.0)	1.0 (1.0–1.0)	
Device size (mm)	28.7 ± 4.1	29.6 ± 3.3	0.479
Device compression (%)	19.8 ± 5.9	19.1 ± 5.3	0.714
Peri-device leak at implantation			
Complete occlusion	19 (86.4)	13 (92.9)	0.546
Leak ≤ 5 mm	3 (13.6)	1 (7.1)	0.546
Leak > 5 mm	0 (0.0)	0 (0.0)	
Procedure-related complications			
Mortality	0 (0.0)	0 (0.0)	
Pericardial effusion	1 (4.5)	0 (0.0)	
Stroke	0 (0.0)	0 (0.0)	
Major bleeding events	0 (0.0)	0 (0.0)	
Complications of vascular access	0 (0.0)	1 (7.1)	

*Values presented are n (%), mean ± standard deviation or median (interquartile range) as appropriate. LAA, left atrial appendage*.

Two patients had procedure-related complications. One patient in the Primary Prevention Group had pericardial effusion, which required percutaneous drainage, and one patient in the Secondary Prevention Group had mild groin hematoma and recovered without surgery.

### Mid-Term Outcomes

All patients underwent scheduled 45-day TEE. At 45 days, TEE showed satisfactory seals in all patients, with complete occlusion in 77.3% of the Primary Prevention Group and 78.6% of the Secondary Prevention Group (Table 3). No patients had residual flow of > 5 mm or DRT. At 6 months, 21 (58.3%) patients underwent a second follow-up TEE and 15 (41.7%) patients underwent a follow-up cardiac CT. Complete seals were identified in 86.4 and 85.7% of the two groups, respectively. One patient in the Secondary Prevention Group revealed asymptomatic DRT by TEE at the 6-month follow-up. The patient was a 75-year-old female with a CHA2DS2-VAS score of 6 and a HAS-BLED score of 3. OAC was discontinued in this patient after the 45-day TEE demonstrated complete occlusion of the LAA. The thrombus was resolved 3 months after reinitiated OAC at the TEE reassessment. Lifelong OAC was recommended for this patient and no thromboembolism or bleeding was recorded afterwards.

**Table 3:** Outcomes at follow-up.

	**Primary prevention *N* = 22**	**Secondary prevention*N* = 14**	***P*-value**
Average follow-up (month)	29.4 ± 10.3	26.7 ± 11.1	0.462
TEE at 45 days follow-up			
Complete occlusion	17 (77.3)	11 (78.6)	0.972
Leak ≤ 5 mm	5 (22.7)	3 (21.4)	0.972
Leak > 5 mm	0 (0.0)	0 (0.0)	
Device-associated thrombosis	0 (0.0)	0 (0.0)	
TEE or CT at 6 months follow-up			
Complete occlusion	19 (86.4)	12 (85.7)	0.956
Leak ≤ 5 mm	3 (13.6)	2 (14.3)	0.956
Leak > 5 mm	0 (0.0)	0 (0.0)	
Device-associated thrombosis	0 (0.0)	1 (7.1)	0.204
OAC off			
At 3 months	18 (81.8)	11 (85.7)	0.760
At 6 months	22 (100.0)	13 (92.9)	0.210
At the end of follow-up	22 (100.0)	13 (92.9)	0.210
Thromboembolic events			
Ischemic stroke/TIA	0 (0.0)	0 (0.0)	
Systemic embolism	0 (0.0)	0 (0.0)	
Major bleeding	1 (4.5)	0 (0.0)	
Mortality	0 (0.0)	0 (0.0)	

*Values presented are n (%) or mean ± standard deviation as appropriate. CT, computed tomography; OAC, oral anticoagulation; TEE, transesophageal echocardiography; TIA, transient ischemic attacks*.

All patients completed at least 12 months of follow-up and the average follow-up was 29.4 ± 10.3 months in the Primary Prevention Group and 26.7 ± 11.1 months in the Secondary Prevention Group (Table 3). There were no thromboembolic events or deaths during follow-up. One patient in the Primary Prevention Group had a bleeding event (gastrointestinal bleeding while on dual antiplatelet therapy of aspirin and clopidogrel) 4 months after the procedure. No bleeding events were reported in the Secondary Prevention Group.

**Figure 1:**
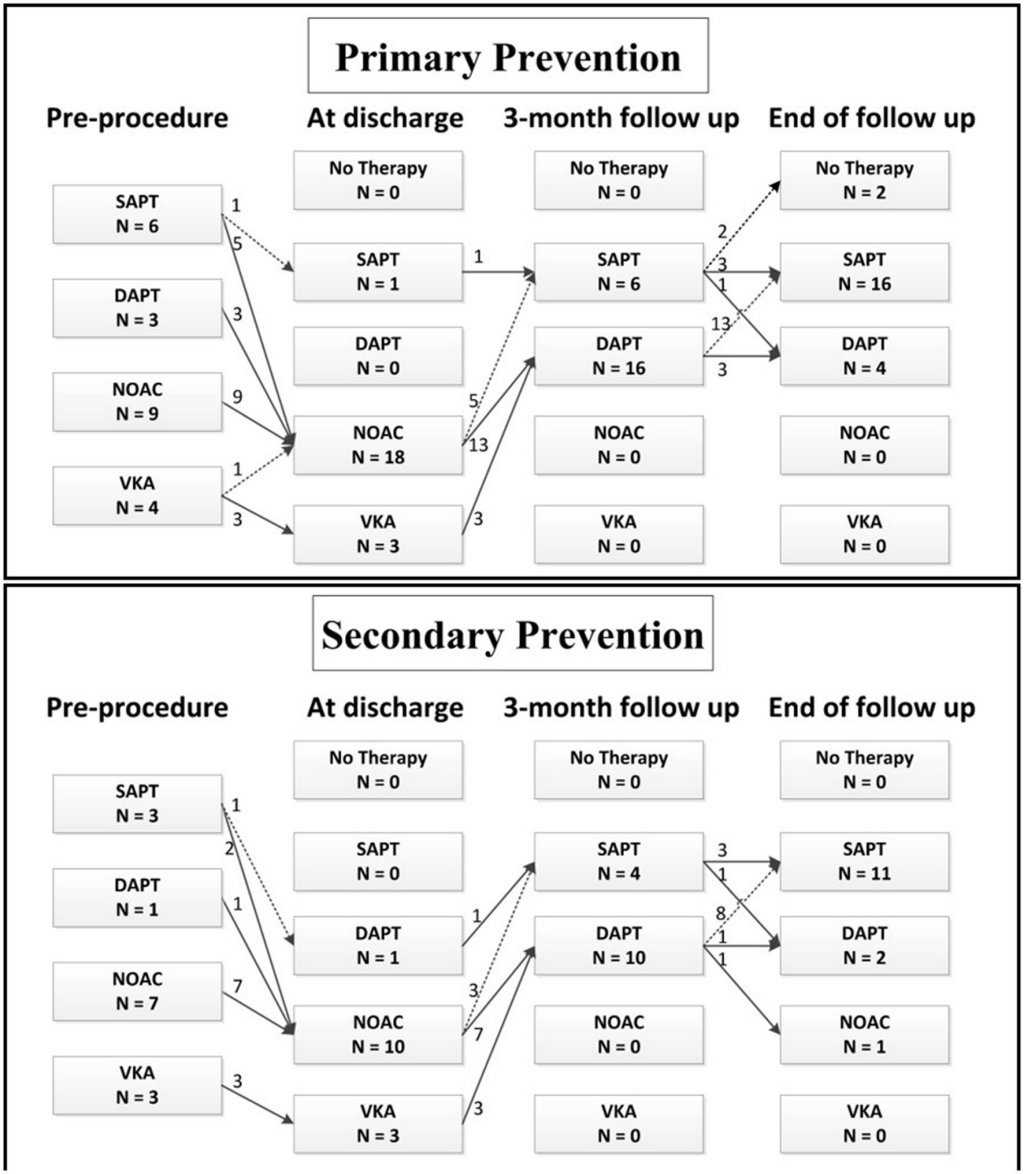
Antithrombotic regimens pre-procedure, at discharge, at 3 months and at the end of follow-up. DAPT, dual antiplatelet therapy; NOAC, novel oral anticoagulation; SAPT, single antiplatelet therapy; VKA, vitamin K antagonist.

The pre- and post-implant antithrombotic treatment is shown in Figure 1. Prior to the procedure, 13 patients (59.1%) in the Primary Prevention Group and 10 (71.4%) in the Secondary Prevention Group received OAC. At 3 months, all patients switched to either dual or single antiplatelet therapy. During the latest follow-up, only one patient (1/36, 2.8%) in the Secondary Prevention Group remained on OAC because of previous DRT. Most of the patients were on single antiplatelet therapy (72.7% in the Primary Prevention Group and 78.6% in the Secondary Prevention Group).

## Discussions

Our pilot study reported the value of LAAC for stroke prevention in patients with HCM and AF. Initial results suggest that LAAC can be a safe and feasible alternative for primary and secondary stroke prevention in selected patients with HCM and AF.

AF is the most common arrhythmia in patients with HCM. The risk of stroke is quite high in patients with concomitant AF and HCM (1, 13). When there was no stroke risk factor other than HCM, the stroke risk of AF patients with HCM was still greater than that of AF patients without HCM with a CHA2DS2-VASc score of 2 (14). Because of the high thromboembolic risk, current guidelines recommend lifelong OAC in all patients with HCM and AF independent of the CHA2DS2-VASc score (3, 4). While warfarin is recommended to prevent thromboembolism in patients with HCM and AF, recent observational data suggest the safety and effectiveness of NOAC in this patient population (15).

Previous studies have shown that >90% of emboli related to non-valvular AF in general population originate from the LAA, leading to the development of mechanical approaches to close the LAA (16, 17). For the past decade, two randomized trials have been conducted and provided evidence for the protective effect of LAAC on thromboembolic events (5, 6). Currently, LAAC is recommend as an alternative to anticoagulation therapy for stroke prevention in the general AF population with high risk of stroke (18). However, the efficacy of LAAC for stroke prevention in the specific population of HCM and AF is unknown.

Gunawardene et al. reported that LAA thrombus was found in 7.1% (2/28) of the patients with AF and HCM by TEE before catheter ablation, but was found in only 0.7% (11/1630) of patients with AF without HCM (7). Another study enrolled a large sample of 205 Asian patients with HCM and AF who had undergone TEE before cardioversion or catheter ablation (8). LAA thrombus was found in 10.7% (22/205) of the patients with HCM. In that study, the incidences of LAA thrombus in patients with HCM and AF with CHA2DS2-VASc scores of 0, 1, and ≥2 were 8.8% (3/34), 9.6% (5/52), and 11.8% (11/119), respectively. These evidences suggest that the high rate of LAA thrombus might explain the high thromboembolic risk in patients with HCM and AF, especially in patients with CHA2DS2-VASc scores of 0 and 1. LAAC in patients with concomitant HCM and AF might therefore provide similar stroke prevention as in the general AF population.

Data of LAAC for primary or secondary stroke prevention in patients HCM and AF is quite limited. Only one case of LAAC in an patient with HCM and paroxysmal AF who suffered repeatedly ocular hemorrhage under OAC was reported (19). We reported initial results from 36 patients with HCM and AF who underwent successful LAAC in this study. Our results showed good periprocedural safety for high-volume operators with low procedural complication rates similar to those in the general AF population in the same center (11). All the patients except one achieved freedom from OAC therapy, with no recorded thromboembolic events during more than 2 years of follow-up in either the primary prevention or the secondary prevention purpose. Our work provides initial evidence for LAAC in primary and secondary stroke prevention in patients with HCM and AF.

### Limitations

This study has several limitations. This is a single-center retrospective study with a small sample size due to the low morbidity of HCM, which is one of the main limitations. Nevertheless, considering the lack of studies investigating LAAC in stroke prevention in the HCM population, this study offers valuable evidence on the use of LAAC for primary and secondary prevention of stroke. Patients with HCM are quite heterogeneous. Sudden cardiac death is more common in younger patients aged 10–35 years, heart failure death is more common in middle-aged patients, and stroke due to HCM-related AF is more common in older patients. Patients with HCM in this study were highly selective, mainly with old age, non- or mild obstruction and without prior surgery or ventricular aneurysm. Therefore, these selected patients had low risk of sudden cardiac death but an increased risk of stroke, and so did not represent the entire population of patients with HCM. Besides, the indications for most patients in the present study were unwilling to receive OAC therapy or having a high bleeding risk. How to select patients with HCM and AF who can benefit from LAAC therapy remains an open question. Further studies are needed to confirm the results of this study.

## Conclusions

We report single center initial results on LAAC for primary and secondary stroke prevention in patients with HCM and AF. Our data suggest that LAAC operations seem to be feasible and safe for stroke prevention in this special population. Further studies with larger samples are required.

## Data Availability Statement

The raw data supporting the conclusions of this article will be made available by the authors, without undue reservation.

## Ethics Statement

The studies involving human participants were reviewed and approved by the Ethics Committee of Xinhua Hospital Affiliated to Shanghai Jiao Tong University School of Medicine. The patients/participants provided their written informed consent to participate in this study.

## Author Contributions

Q-SW and Y-GL were in charge of the design of this study. B-FM and RZ wrote the manuscript. All authors contributed to the data collection, analyses, and approved the final manuscript.

## Conflict of Interest

The authors declare that the research was conducted in the absence of any commercial or financial relationships that could be construed as a potential conflict of interest.

## Publisher's Note

All claims expressed in this article are solely those of the authors and do not necessarily represent those of their affiliated organizations, or those of the publisher, the editors and the reviewers. Any product that may be evaluated in this article, or claim that may be made by its manufacturer, is not guaranteed or endorsed by the publisher.

## Abbreviations

AF: atrial fibrillation
CT: computed tomography
DRT: device-related thrombus
HCM: hypertrophic cardiomyopathy
LAA: left atrial appendage
LAAC: left atrial appendage closure
NOAC: novel oral anticoagulant
OAC: oral anticoagulation
TEE: transesophageal echocardiography.
